# Predictive value of De Ritis ratio in metastatic renal cell carcinoma treated with tyrosine-kinase inhibitors

**DOI:** 10.1007/s00345-021-03628-2

**Published:** 2021-03-01

**Authors:** Florian Janisch, Thomas Klotzbücher, Phillip Marks, Christina Kienapfel, Christian P. Meyer, Hang Yu, Constantin Fühner, Tobias Hillemacher, Keiichiro Mori, Hadi Mostafei, Shahrokh F. Shariat, Margit Fisch, Roland Dahlem, Michael Rink

**Affiliations:** 1 Department of Urology, University Medical Center Hamburg-Eppendorf, Martinistraße 52, 20246 Hamburg, Germany; 2 Department of Urology, Medical University of Vienna, Vienna, Austria; 3 Department of Urology, Jikei University School of Medicine, Tokyo, Japan; 4 Department of Urology, Tabriz University of Medical Sciences, Tabriz, Iran; 5 Institute for Urology and Reproductive Health, Sechenov University, Moscow, Russia; 6 Department of Urology, Weill Cornell Medical School, New York, NY USA; 7 Department of Urology, University of Texas Southwestern Medical Center, Dallas, TX USA; 8 Karl Landsteiner Institute of Urology and Andrology, Vienna, Austria; 9 Department of Urology, Second Faculty of Medicine, Charles University, Prague, Czech Republic

**Keywords:** Metastatic renal cell carcinoma, Tyrosine-kinase inhibitors, Tumor markers, Prognostic marker, De ritis ratio

## Abstract

**Background:**

Predictive markers can help tailor treatment to the individual in metastatic renal cell carcinoma (mRCC). De Ritis ratio (DRR) is associated with oncologic outcomes in various solid tumors.

**Objective:**

To assess the value of DRR in prognosticating survival in mRCC patients treated with tyrosine-kinase inhibitors (TKI).

**Methods:**

Overall, 220 mRCC patients treated with TKI first-line therapy were analyzed. An optimal cut-off point for DRR was determined with Youden’s J. We used multiple strata for DRR, performed descriptive, Kaplan–Meier and multivariable Cox-regression analyses to assess associations of DRR with progression-free (PFS) and overall survival (OS).

**Results:**

Patients above the optimal cut-off point for DRR of ≥ 1.58 had fewer liver metastases (*p* = 0.01). There was no difference in PFS (*p* > 0.05) between DRR groups. DRR above the median of 1.08 (HR 1.42; *p* = 0.03), DRR ≥ 1.1(HR 1.44; *p* = 0.02), ≥ 1.8 (HR 1.56; *p* = 0.03), ≥ 1.9 (HR 1.59; *p* = 0.02) and ≥ 2.0 (HR 1.63; *p* = 0.047) were associated with worse OS. These associations did not remain after multivariable adjustment. In the intermediate MSKCC group, DRR was associated with inferior OS at cut-offs ≥ 1.0 (HR 1.78; *p* = 0.02), ≥ 1.1 (HR 1.81; *p* = 0.01) and above median (HR 1.88; *p* = 0.007) in multivariable analyses. In patients with clear-cell histology, DRR above median (HR 1.54; *p* = 0.029) and DRR ≥ 1.1 (HR 1.53; *p* = 0.029) were associated with OS in multivariable analyses.

**Conclusion:**

There was no independent association between DRR and survival of mRCC patients treated with TKI in the entire cohort. However, OS of patients with intermediate risk and clear-cell histology were affected by DRR. DRR could be used for tailored decision-making in these subgroups.

**Supplementary Information:**

The online version contains supplementary material available at 10.1007/s00345-021-03628-2.

## Introduction

Despite the progress in the therapy of metastatic renal cell carcinoma (mRCC), including the introduction of tyrosine-kinase inhibitors (TKI), survival rates for mRCC remain low with < 20% of patients surviving beyond 5 years from their diagnosis [1]. Currently used prognostic risk stratifications such as the Memorial Sloan Kettering Cancer Center (MSKCC) and the International Metastatic RCC Database Consortium (IMDC) [2, 3] have great value in identifying patients’ prognosis. However, the rapid evolution of systemic treatment and surge of combination therapies requires further individualized refinement of treatment to provide optimal treatment for mRCC patients.

To address this difficulty, several prognostic markers have been investigated to help daily clinical decision-making and tailoring treatment to the individual [4, 5]. However, as of now, none of the investigated biomarkers pass from bench-to-bedside [6].

Inflammatory markers have been suggested to provide important information about the function of our body’s immune system with carcinomas. Multiple actors in the immune system such as the C-reactive protein and neutrophil-to-lymphocyte ratio have been shown to be of prognostic relevance in mRCC [7]. The De Ritis ratio (DRR), the ratio between the serum concentrations of aspartate transaminase (AST) and alanine transaminase (ALT), demonstrated to predict survival outcomes in hepatic disease as well as other solid organ malignancies [8, 9]. Recent reports found the DRR to be of value in patients with localized RCC [10]. DRR is an easily assessable and cheap marker that could prove valuable in the prognostication of mRCC. However, specific cut-off points for DRR are not well defined and possibly differ from disease state, type and population case mix.

We hypothesized that the DRR is a prognosticator for oncologic outcomes in patients with mRCC. The aim of this study is to analyze the association between DRR and different survival endpoints in a consecutive, real-world cohort of mRCC patients treated with TKI therapy.

## Materials and methods

### Patient selection

We retrospectively reviewed the records of 398 consecutive patients with mRCC treated at our institution from 2006 to 2016. Only patients with primary TKI therapy with or without cytoreductive nephrectomy were included in this study. Patients with prior immunotherapy, missing follow-up, clinical information or laboratory values, or proven liver disease prior to treatment were excluded (*n* = 178). This left a total of 220 patients for analysis. Data for baseline characteristics and clinical outcome including age, gender, Eastern Cooperative Oncology Group performance status (ECOG), underlying primary histology, pathological T-stage, nodal status, details about performed cytoreductive nephrectomy, number and locations of metastasis, presence of secondary malignancy in patient’s history, metastasectomy and therapy lines were gathered. The MSKCC risk score was calculated for each patient [3]. ECOG status, MSKCC and Karnofsky score were assessed before treatment. ALT and AST were assessed before initiation of systemic therapy or cytoreductive nephrectomy. Cut-off of metastatic locations was ≤ 2 vs. ≥ 3 metastatic sites, as previously described [11]. The study was approved by the institutional review board.

### Histological assessment

Surgical specimens were processed according to standard pathologic procedures. Tumor stage and grade were classified according to the American Joint Committee on Cancer–Union Internationale Contre le Cancer TNM classification and the 1998 WHO/International Society of Urologic Pathology consensus classification. Incomplete pathological stage or nodal status due to specimen taken from biopsies was categorized as pTx/pNx. Primary histology was assessed by a dedicated uro-pathologist according to the World Health Organisation (WHO) histologic classification. Histology was grouped according to subtypes in clear-cell, papillary, chromophobe and “other” RCC, respectively [12]. Sarcomatoid histology was assessed from the primary tumor or biopsy, as previously described [13].

### Follow-up

Patients were regularly seen in our outpatient clinic and the follow-up was performed according to the current guidelines at the time [11]. Clinical examination and laboratory controls were performed monthly. Diagnostic imaging of the abdomen and pelvis as well as chest radiography were conducted quarterly. Additional radiographic evaluations (e.g., bone or brain imaging) were performed when clinically indicated. Primary co-endpoints were progression-free (PFS) and overall survival (OS). Disease progression was defined by clinical progression according to the current Response Evaluation Criteria in Solid Tumors (RECIST) version at the time of evaluation [14]. All patients without disease progression or death at last follow-up were censored.

### Statistical analysis

Continuous variables were reported as mean and standard error when normal distributed, or as median and interquartile range. DRR score was calculated as AST divided by ALT. Youden’s J statistic was used to determine the optimal cut-off point for the DRR ratio with a receiver-operator curve using median cancer-specific survival as an end-point [15, 16]. In addition, the DRR ratio was analyzed as a continuous variable, stratified at the median, tertiles and according to cut-off points from 1.0 to 2.0 in 0.1 steps to account for possible intervariability of study cohorts in the current literature and provide the most complete evidence. Restricted cubic splines were used to visualize the relation between DRR and the probability of death [17]. Baseline characteristics were compared using the Chi-square test or Fisher’s exact test for nominal variables and Student’s *T* test or Mann–Whitney *U* test for continuous variables. Survival analysis was performed with Kaplan–Meier estimates using the log-rank test for pairwise comparison. Uni- and multi-variable analyses were performed with a Cox-regression model, adjusting for the effect of clinically relevant factors. We performed a competing risk analysis using cancer-specific and other-cause mortality. All analyses were performed in STATA 14.0 (Stata Corp., College Station, TX). Statistical results were considered significant if the *p* value was < 0.05; all tests were two sided.

## Results

### Baseline characteristics

Youden’s J revealed an optimal cut-off point for DRR at 1.58 (supplementary Fig. 1); patients were grouped accordingly into “low” and “high DRR”, and baseline characteristics are displayed in Table 1 accordingly. Overall, 171 patients (77.7%) had a low DRR and 49 (22.3%) had a high DRR. Median AST was 22 IU/L (IQR 17;33 IU/L) and median ALT was 21 IU/L (IQR 14;32 IU/L) resulting in a median DRR of 1.08 (IQR 0.81–1.50). Median age was 64 years (IQR 57;71) and 163 patients (74.1%) were male. Most patients had clear-cell histology (*n* = 182; 82.7%) and received Sunitinib as first-line treatment (*n* = 153; 69.6%), respectively. 157 (71.4%) patients had ≥ 3 metastatic sites and 73 (33.2%) had liver metastases. Patients with high DRR had less frequent liver metastasis compared to those with low DRR (*p* = 0.01), but no difference in the number of metastatic sites. There were no differences in other baseline characteristics between the two groups.

**Table 1:** Baseline characteristics of patients with metastatic renal cell carcinoma treated with tyrosine-kinase inhibitors stratified by DRR

n (%)	Overall	Low DRR^b^ 171 (77.7)	High DRR^b^ 49 (22.3)	*p* value
	220			
Age—median (IQR)	64 (57–71)	64 (57–71)	63 (59–70)	0.8
Male gender	163 (74.1)	126 (73.7)	31 (75.1)	0.06
ECOG ≥ 2	27 (12.3)	20 (11.8)	7(14.3)	0.63
Karnofsky > 80%	152 (69.4)	123 (72.4)	29 (59.2)	0.08
*Histology*				0.6
Clear cell	182 (82.7)	143 (83.6)	39 (79.6)	
Papillary	28 (12.7)	22 (12.9)	6 (12.2)	
Chromophobe	7 (3.2)	4 (2.3)	3 (6.1)	
Other	3 (1.4)	2 (1.2)	1 (2.0)	
Variant histology	18 (8.2)	15 (8.8)	3 (6.1)	0.6
Sarcomatoid component	16 (7.3)	14 (8.2)	2(4.1)	0.3
*pT stage*				0.6
pT1	51 (23.2)	42 (24.6)	9 (18.4)	
pT2	35 (15.9)	28 (16.4)	7 (14.3)	
pT3	92 (41.8)	72 (42.1)	20 (40.8)	
pT4	14 (6.4)	10 (5.9)	4 (8.2)	
pTx^a^	28 (12.7)	19 (11.1)	9 (18.4)	
*Nodal status*				0.4
pN0	79 (35.9)	65 (38.0)	14 (28.6)	
pN +	43(19.6)	31 (18.3)	23 (46.9)	
pNx^a^	98 (44.5)	75 (43.9)	12 (24.5)	
*MSKCC score*				0.4
Good	47 (21.4)	36 (21.1)	11 (22.5)	
Intermediate	128 (58.2)	103 (60.2)	25 (51.0)	
Poor	45 (20.5)	32 (18.7)	13 (26.5)	
Secondary malignancy	35 (15.9)	24 (14.0)	11 (22.5)	0.16
AST—median (IQR)	22 (17–33)	22 (17–32)	24 (18–33)	0.6
ALT—median (IQR)	21 (14–32)	24 (17–38)	12 (9–18)	** < 0.001**
DRR—median (IQR)	1.08 (0.81–1.50)	0.94 (0.73–1.21)	1.92 (1.75–2.12)	** < 0.001**
*Metastasis locations*				
Brain	33 (15.0)	23 (13.5)	10 (20.4)	0.2
Bone	113 (51.4)	84 (59.1)	29 (59.2)	0.2
Lung	162 (73.6)	124 (72.5)	38 (77.6)	0.5
Liver	73 (33.2)	64 (37.4)	9 (18.4)	**0.01**
Lymph nodes	163 (74.1)	124 (72.5)	39 (79.6)	0.3
Other	127 (57.7)	99 (57.9)	28 (57.1)	0.9
≥ 3 metastastic sites	157 (71.4)	125 (73.1)	32 (65.3)	0.3
Cytoreductive nephrecotmy	83 (37.7)	65 (38.0)	18 (36.7)	0.9
Metastasectomy	114 (51.8)	86 (50.3)	28 (57.1)	0.4
*First-line therapy*				0.4
Suntinib	153 (69.6)	122 (71.4)	31 (63.3)	
Sorafenib	29 (13.1)	20 (11.7)	5 (10.2)	
Pazopanib	27 (12.3)	22 (12.9)	9 (18.4)	
Other	11 (5.0)	7 (4.1)	4 (8.2)	
≥ 3 therapy lines	63 (28.6)	53 (31.0)	10 (20.4)	0.1

Bold values indicate significant (*p* < 0.05)

*ALT* alanine transaminase, *AST* aspartate transaminase, *DRR* De Ritis ratio (AST/ALT), *ECOG* Eastern Cooperative Oncology Group performance status, *MSKCC* Memorial Sloan Kettering Cancer Center, *IQR* interquartile range, *pT stage* pathological tumor stage

^a^Pathological nodal status /T stage not available in patients with biopsies only

^b^Cut-off point for high and low De Ritis ratio 1.58

### Association of DRR with survival outcomes

**Fig. 1:**
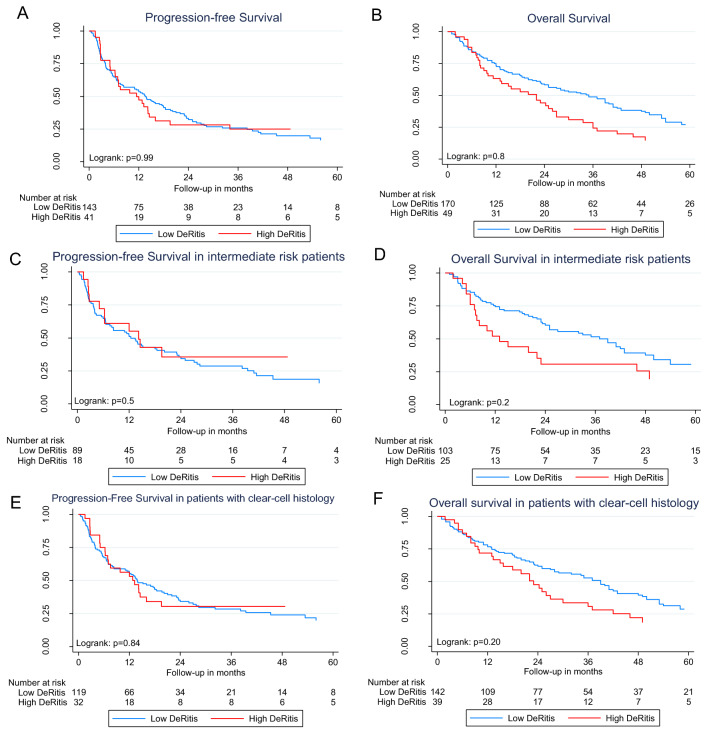
Kaplan–Meier estimates of progression-free (**a**) and overall survival (**b**) of patients with metastatic renal cell carcinoma and the subgroups of intermediate-risk patients (progression-free survival; **c**) (overall survival; **d**) and clear-cell histology (progression-free survival; **e**) (overall survival; **f**) stratified by a low (< 1.58) and high (≥ 1.58) De Ritis ratio

The median follow-up was 72 months (IQR 42;126), median PFS was 13 months (IQR 4;38) and median OS was 28 months (IQR 10;58). Median observed and expected mortalities in relation to DRR are presented in supplementary Fig. 2 with the probability of death rising from a low DRR to 1.8 and a falling with a low decrement at higher levels. Kaplan–Meier estimates did not reveal a difference in PFS between patients with high or low DRR (Fig. 1a). There was also no association between DRR and PFS for all analyzed strata (all *p* > 0.05; Table 2).

**Table 2:** Uni- and multi-variable regression analyses of different strata for De Ritis ratio (DRR) and its impact on progression-free (PFS) and overall survival (OS)

	Univariable PFS			Univariable OS			Multivariable OS		
	HR	95% CI	*p* value	HR	95% CI	*p* value	HR	95% CI	*p* value
DRR (continuous)	1.06	0.84–1.33	0.64	1.22	0.98–1.52	0.08	–	–	–
High vs. low DRR^a^	1.00	0.67–1.50	0.99	1.37	0.96–1.96	0.08	–	–	–
DRR ≥ median vs. < median	1.11	0.80–1.55	0.54	1.42	1.04–1.93	**0.03**	1.37	0.99–1.93	0.07
*DRR tertiles*									
2nd vs. 1st	1.33	0.88–2.02	0.18	1.21	0.92–1.77	0.34	–	–	–
3rd vs. 1st	1.16	0.77–1.75	0.48	1.31	0.90–1.91	0.16	–	–	–
DRR ≥ 1.1 vs. < 1.1	1.05	0.75–1.46	0.79	1.44	1.06–1.96	**0.02**	1.36	0.97–1.90	0.07
DRR ≥ 1.8 vs. < 1.8	1.18	0.75–2.87	0.47	1.56	1.05–2.31	**0.03**	1.49	0.99–2.26	0.056
DRR ≥ 1.9 vs. < 1.9	1.25	0.76–2.06	0.38	1.59	1.04−2.44	**0.03**	1.37	0.87–2.15	0.18
DRR ≥ 2.0 vs. < 2.0	1.29	0.73–2.29	0.39	1.63	1.01–2.64	**0.047**	1.30	0.78–2.17	0.31

Bold values indicate significant (*p* < 0.05)

All DRR cut-off points that were not significant in the univariable analysis are not shown

Multivariable analysis adjusted for significant values in the univariable analysis of the following: Eastern Cooperative Oncology Group performance status (ECOG), histology subgroup and sarcomatoid features, Memorial Sloan Kettering Cancer Center prognostic risk score (MSKCC), number of therapy lines, presence of sarcomatoid histology, secondary malignancy, T stage and lymph node metastasis, presence of liver metastasis and number of metastatic locations

Adjustments in multivariable analyses were performed for ECOG histology subgroup and sarcomatoid features, T stage and lymph node metastasis, MSKCC, and number of therapy lines for PFS and for ECOG, sarcomatoid histology, cytoreductive nephrectomy, pathological T stage and lymph node metastasis, MSKCC for OS, respectively

^a^Cut-off point for high and low De Ritis ratio 1.58

Patients with low DRR had better OS in Kaplan–Meier estimates (35 vs. 22 months), which was not statistically significant (*p* = 0.08; Fig. 1b). A high DRR, continuous DRR, and DRR stratified by tertiles (cut-off points: 0.89 and 1.36) were not significantly associated with OS in univariable analyses (Table 2). Patients with DRR above the median compared to those with DRR below median (HR 1.42; 95% CI = 1.04–1.93; *p* = 0.03), and all DRR above the threshold of 1.1 (HR 1.44; 95% CI = 1.06–1.96; *p* = 0.02), 1.8 (HR 1.56; 95% CI = 1.05–2.31; *p* = 0.03), 1.9 (HR 1.59; 95% CI = 1.06–1.96; *p* = 0.03) and 2.0 (HR 1.63; 95% CI = 1.01–2.64; *p* = 0.047) were associated with worse OS compared to patients below these cut-off points (Table 2). These associations, however, did not remain significant in multivariable analyses that adjusted for standard outcome mRCC parameters (all *p* > 0.05, Table 2). All other cut-off points for DRR did not show any association with survival outcomes in univariable analyses. Competing risk analysis showed no difference between high DRR and low DRR for cancer-specific and other-cause mortality (Supplementary Fig. 3; *p* > 0.05).

### Subgroup analyses of prognostic risk groups

The results of subgroup analyses are presented in supplementary Table 1 and Fig. 1c, d. In patients with good prognosis, there was no association between all strata for DRR and PFS or OS in multivariable analyses (all *p* > 0.05). In the subgroup of patients with intermediate prognosis, PFS was not associated with DRR (all *p* > 0.05). DRR higher than the cut-off points of 1.0 (HR 1.78; 95% CI = 1.09–2.90; *p* = 0.02) and 1.1 (HR 1.81; 95% CI = 1.15–2.85; *p* = 0.01), as well as the median (HR 1.88; 95% CI = 1.19–2.96; *p* = 0.007) were associated with inferior OS in multivariable analyses in patients with intermediate prognosis. All other cut-off points for DRR did not show any association with survival outcomes in univariable analyses. In poor prognosis patients, PFS was only associated with continuous DRR (HR 0.63; 95% CI = 0.39–1.00; *p* = 0.049) in univariable analysis. This effect, however, did not retain in multivariable analysis (*p* > 0.05). There was no association between DRR and OS in poor prognosis patients.

### Subgroup analyses of patients with clear-cell histology

Supplementary Table 2 and Fig. 1e, f show the results from the subgroup analyses of patients with and without clear-cell histology. In patients with clear-cell histology, there was no association between PFS and DRR. Patients with DRR higher than the cut-off points 1.1 (HR 1.53; 95% CI = 1.04–2.24; *p* = 0.029) as well as the median (HR 1.54; 95% CI = 1.05–2.29; *p* = 0.029) had shorter OS compared to those below these thresholds in multivariable analyses. There was no association between either PFS or OS and DRR in patients with non-clear-cell histology (all *p* > 0.05).

## Discussion

In this study, we analyzed the association between DRR and oncologic outcomes in mRCC patients treated with TKI. The cut-off point determined by Youden’s J could not prove more predictive for survival in comparison to other strata. We found no difference in PFS or OS in patients with higher DRR, regardless of cut-off points or stratification method. Nonetheless, competing risk analysis did not show any difference in cancer-specific survival in patients with high DRR. Thus, our findings challenge the value of DRR as a prognostic marker in mRCC patients.

Indeed, our results are in contrast to some body of literature. Kang et al. reported better OS and CSS with DRR < 1.2 before TKI therapy initiation [18]. Their patients with higher DRR, however, were significantly older, and > 20% of underlying histology and pathological T stage was missing from their dataset, which possibly skewed results. Kim et al. found higher continuous DRR to be an independent factor for worse OS, but not PFS in patients with mRCC [19]. However, the study included patients with missing AST or ALT as well, possibly introducing bias. Another study reported that DRR ≥ 1.24 was associated with worse OS and CSS in patients with mRCC that underwent cytoreductive nephrectomy [20]. In a recent meta-analysis, DRR was associated with survival in patients with RCC [8]. However, the heterogeneous designs of the included studies, including localized and metastatic disease as well as different cut-off points, impair comparability. Interestingly, we found that patients with clear-cell histology had worse OS when DRR was ≥ 1.1 or the median of 1.08. Patients with other, varied underlying histologic subtypes might have skewed results in the entire cohort. In contrast, Sekar et al. found no difference in OS in their subgroup analyses of patients with clear-cell histology [21]. However, this study included all patients with performed nephrectomy and included patients with localized and metastatic disease. This further suggests that heterogeneity in study cohorts should be minimized to shed light on optimal cut-off points for DRR and help finding suitable patient subgroups to incorporate this marker into clinical treatment. Thus, our findings are of utmost importance as the true value of DRR in mRCC should be reevaluated in larger and ideally prospective studies.

We found that patients in the MSKCC intermediate-risk group with DRR lower than the cut-off point 1.0–1.1 had improved OS, while outcomes were not influenced in other risk groups in multivariable analysis. These findings warrant consideration, as prognostic risk group scores are standard tools in mRCC for patient stratification to facilitate optimal systemic treatment. However, particularly the intermediate-risk group, which comprises the largest number of patients, is challenging regarding treatment and prognosis, due to its heterogeneity. Indeed, differential survival outcomes in this risk group are highly dependent on the number of risk factors [22]. Patients with only one risk factor in the intermediate-risk group according to IMDC score had a 13-month-longer median OS in mRCC patients treated with sunitinib [23]. This underscores the necessity of differential assessment in the intermediate-risk group. Further, insignificant results of the good and poor prognosis groups should be validated, to overcome potential impairment by small sample sizes. Incorporation of DRR into established risk scores may have the potential to tailor patient counseling and help adjust follow-up regimen. Additional research in larger, prospective studies is recommended, as the current body of literature remains inconclusive and our results are just hypothesis generating [19, 21].

One of the first studies to describe the impact of DRR on survival outcomes in cancer patients is the study of Bezan et al., which focused on patients with localized RCC [24]. Since then, several other studies investigated the association of DRR and survival in localized RCC with conflicting results [10, 25]. Indeed, in mRCC, the metastatic burden predominantly impacts survival. Visceral metastases, especially liver metastases, are associated with poor oncologic outcomes [26]. High transaminase levels may be used as a surrogate parameter for liver metastasis (i.e., liver damage), and, in consequence, for oncologic outcomes. Therefore, DRR could possibly present an interesting marker depending on metastatic location patterns in contrast to other prognosticators. Albeit, the number of metastatic sites had no association with high DRR in our study. Interestingly, hepatic metastases were even less frequent in patients with a high DRR. To avoid bias, we calculated DRR based on pre-treatment laboratory values, as transaminase levels may be influenced by systemic therapy. Still, the value of this score should be reevaluated during the course of treatment, as visceral metastases may develop during disease progression.

Our study is not without limitations. First, the retrospective single-center design introduces patient selection bias. Second, as not all included patients underwent nephrectomy at any time, information about pathological tumor stage and nodal status was not available for all patients, which may have influenced statistical analyses. Third, metastatic burden was assessed by imaging and not always verified by biopsy. Fourth, DRR is an unspecific marker that could be affected by other comorbidities and drugs as well, despite defining clear in- and exclusion criteria. Additionally, not all comorbidities and corresponding medication were present in our data set in detail, and thus could not be included, which might have influenced our findings. Meanwhile, new drugs have progressed to clinical standard in mRCC [27] and mainly superseded those in our cohort. However, TKI used in our study were standard of care at the time of treatment and, therefore, should not bias outcomes. In addition, TKI still remains an important treatment option in selected patients. Reevaluation of DRR in current treatment regimens including immuno-oncology (IO)/TKI or IO/IO combinations is warranted. Further, a single measurement of DRR before treatment cannot solely portray the dynamics in each patient’s metabolism during systemic therapy. As TKI therapy can result in the elevation of liver enzyme levels, DRR might not be a fitting prognosticator for further therapy lines. While setting a standardized point is necessary for accurate statistics, the prognostic value of DRR might change during systemic therapy. However, we feel that the variable strata used to define cut-off points in our study provided valuable insight on DRR as a prognostic marker.

## Conclusion

There was no strong association between various DRR strata and survival outcomes in our entire mRCC cohort. However, DRR may help in patient prognostication in the large, heterogeneous MSKCC intermediate-risk subgroup as well as in patients with clear-cell histology. Thus, the incorporation of DRR in clinical decision-making and treatment guidance of intermediate-risk patients in our western European population warrants further investigation. Additional, larger, prospective studies are needed to further assess the value of DRR in other western-world regions and address potential confounders.

## Supplementary Information

Below is the link to the electronic supplementary material.

Supplementary file1 (DOCX 27 KB)

Supplementary file2 (DOCX 23 KB)

Supplementary file3 (PDF 49 KB)

Supplementary file4 (PDF 56 KB)

Supplementary file5 (PDF 73 KB)
