# Supplementary material for: Predictive value of De Ritis ratio in metastatic renal cell carcinoma treated with tyrosine-kinase inhibitors
Source: World J Urol. 2021 Mar 1;39(8):2977–85. doi: 10.1007/s00345-021-03628-2 (PMC8405478; doi:10.1007/s00345-021-03628-2)
Supplement: Supplementary file 1 — Supplementary file1 (DOCX 27 KB) [file 345_2021_3628_MOESM1_ESM.docx]

| Supplementary Table 1: Subgroup analysis according to Memorial Sloan Kettering Cancer Center scores of different strata for De Ritis ratio (DRR) and its impact on progression-free (PFS) and overall survival (OS) | | | | | | |
| --- | --- | --- | --- | --- | --- | --- |
| **Good prognosis** | | | | | | |
|  | **Univariable PFS** | | | **Univariable OS** | | |
|  | HR | HR | HR | HR | 95%CI | P-value |
| **High DRR*** | 1.33 | 1.33 | 1.33 | 1.82 | 0.58-3.04 | 0.50 |
| **DRR (continuous)** | 1.40 | 1.40 | 1.40 | 1.69 | 0.67-2.93 | 0.37 |
| **DRR ≥1.0 vs. <1.0** | 1.30 | 1.30 | 1.30 | 0.90 | 0.60-2.84 | 0.50 |
| **DRR ≥1.1 vs. <1.1** | 0.94 | 0.94 | 0.94 | 1.46 | 0.45-1.96 | 0.87 |
| **DRR ≥1.2 vs. <1.2** | 1.01 | 1.01 | 1.01 | 1.95 | 0.48-2.12 | 0.98 |
| **DRR ≥1.3 vs. <1.3** | 1.00 | 1.00 | 1.00 | 1.55 | 0.47-2.14 | 1.00 |
| **DRR ≥1.4 vs. <1.4** | 1.05 | 1.05 | 1.05 | 1.38 | 0.49-2.25 | 0.90 |
| **DRR ≥1.5 vs. <1.5** | 1.35 | 1.35 | 1.35 | 2.04 | 0.62-2.91 | 0.45 |
| **DRR ≥1.6 vs. <1.6** | 1.33 | 1.33 | 1.33 | 1.82 | 0.58-30.4 | 0.50 |
| **DRR ≥1.7 vs. <1.7** | 1.12 | 1.12 | 1.12 | 1.45 | 0.45-2.79 | 0.82 |
| **DRR ≥1.8 vs. <1.8** | 1.65 | 1.65 | 1.65 | 2.21 | 0.67-4.10 | 0.28 |
| **DRR ≥1.9 vs. <1.9** | 1.11 | 1.11 | 1.11 | 1.83 | 0.38-3.20 | 0.85 |
| **DRR ≥2.0 vs. <2.0** | 0.90 | 0.90 | 0.90 | 1.23 | 0.21-3.83 | 0.88 |
| **DRR ≥median vs. <median** | 1.14 | 1.14 | 1.14 | 1.22 | 0.54-2.38 | 0.73 |
| **DRR ≥first tertile vs. <second tertile** | 2.39 | 2.39 | 2.39 | 0.97 | 0.89-6.41 | 0.08 |
| **DRR ≥first tertile vs. <third tertile** | 1.73 | 1.73 | 1.73 | 1.36 | 0.63-4.72 | 0.29 |
| **Intermediate prognosis** | | | | | | |
|  | **Univariable PFS** | | | **Univariable OS** | | |
|  | HR | HR | HR | HR | 95%CI | P-value |
| **High DRR*** | 0.79 | 1.39 | 1.39 | 1.39 | 0.43-1.47 | 0.46 |
| **DRR (continuous)** | 1.18 | 1.42 | 1.42 | 1.42 | 0.83-1.29 | 0.35 |
| **DRR ≥1.0 vs. <1.0** | 1.26 | 1.91 | 1.91 | 1.91 | 0.81-1.97 | 0.31 |
| **DRR ≥1.1 vs. <1.1** | 1.11 | 1.86 | 1.86 | 1.86 | 0.72-1.74 | 0.63 |
| **DRR ≥1.2 vs. <1.2** | 0.93 | 1.42 | 1.42 | 1.42 | 0.59-1.47 | 0.77 |
| **DRR ≥1.3 vs. <1.3** | 0.91 | 1.33 | 1.33 | 1.33 | 0.57-1.46 | 0.69 |
| **DRR ≥1.4 vs. <1.4** | 0.92 | 1.28 | 1.28 | 1.28 | 0.57-1.50 | 0.74 |
| **DRR ≥1.5 vs. <1.5** | 0.81 | 1.21 | 1.21 | 1.21 | 0.47-1.38 | 0.43 |
| **DRR ≥1.6 vs. <1.6** | 0.79 | 1.39 | 1.39 | 1.39 | 0.43-1.47 | 0.46 |
| **DRR ≥1.7 vs. <1.7** | 0.82 | 1.34 | 1.34 | 1.34 | 0.43-1.56 | 0.55 |
| **DRR ≥1.8 vs. <1.8** | 1.00 | 1.51 | 1.51 | 1.51 | 0.50-2.02 | 0.99 |
| **DRR ≥1.9 vs. <1.9** | 1.55 | 1.76 | 1.76 | 1.76 | 0.74-3.23 | 0.25 |
| **DRR ≥2.0 vs. <2.0** | 1.88 | 2.18 | 2.18 | 2.18 | 0.81-4.36 | 0.14 |
| **DRR ≥median vs. <median** | 1.16 | 1.92 | 1.92 | 1.92 | 0.74-1.80 | 0.52 |
| **DRR ≥first tertile vs. <second tertile** | 1.35 | 1.59 | 1.59 | 1.59 | 0.79-2.31 | 0.28 |
| **DRR ≥first tertile vs. <third tertile** | 1.11 | 1.66 | 1.66 | 1.66 | 0.65-1.92 | 0.70 |
|  | **Multivariable PFS** | | | **Multivariable OS** | | |
|  | HR | 95%CI | P-value | HR | 95%CI | P-value |
| **DRR (continuous)** | / | / | / | 1.37 | 0.95-1.97 | 0.09 |
| **DRR ≥1.0 vs. <1.0** | / | / | / | 1.78 | 1.09-2.90 | **0.02** |
| **DRR ≥1.1 vs. <1.1** | / | / | / | 1.81 | 1.15-1.85 | **0.01** |
| **DRR ≥2.0 vs. <2.0** | / | / | / | 2.21 | 0.96-5.11 | 0.06 |
| **Poor prognosis** | | | | | | |
|  | **Univariable PFS** | | | **Univariable OS** | | |
|  | HR | 95%CI | P-value | HR | 95%CI | P-value |
| **High DRR*** | 0.72 | 0.33-1.59 | 0.42 | 0.98 | 0.50-1.93 | 0.95 |
| **DRR (continuous)** | **0.63** | **0.39-0.99** | **0.049** | 0.79 | 0.52-1.20 | 0.26 |
| **DRR ≥1.0 vs. <1.0** | 0.62 | 0.30-1.30 | 0.21 | 0.59 | 0.31-1.11 | 0.10 |
| **DRR ≥1.1 vs. <1.1** | 0.79 | 0.38-1.65 | 0.54 | 0.71 | 0.39-1.32 | 0.28 |
| **DRR ≥1.2 vs. <1.2** | 0.70 | 0.34-1.47 | 0.35 | 0.78 | 0.42-1.44 | 0.42 |
| **DRR ≥1.3 vs. <1.3** | 0.70 | 0.34-1.47 | 0.35 | 0.71 | 0.38-1.32 | 0.27 |
| **DRR ≥1.4 vs. <1.4** | 0.78 | 0.36-1.69 | 0.53 | 1.08 | 0.56-2.10 | 0.72 |
| **DRR ≥1.5 vs. <1.5** | 0.72 | 0.33-1.59 | 0.42 | 0.98 | 0.50-1.93 | 0.95 |
| **DRR ≥1.6 vs. <1.6** | 0.72 | 0.33-1.59 | 0.42 | 0.98 | 0.50-1.93 | 0.95 |
| **DRR ≥1.7 vs. <1.7** | 0.72 | 0.29-1.77 | 0.47 | 1.08 | 0.51-2.30 | 0.83 |
| **DRR ≥1.8 vs. <1.8** | 0.72 | 0.29-1.77 | 0.47 | 1.08 | 0.51-2.30 | 0.83 |
| **DRR ≥1.9 vs. <1.9** | 0.60 | 0.23-1.59 | 0.31 | 1.00 | 0.46-2.19 | 1.00 |
| **DRR ≥2.0 vs. <2.0** | 0.48 | 0.17-1.40 | 0.18 | 0.88 | 0.39-2.00 | 0.75 |
| **DRR ≥median vs. <median** | 0.62 | 0.30-1.30 | 0.21 | 0.59 | 0.31-1.11 | 0.10 |
| **DRR ≥first tertile vs. <second tertile** | 0.91 | 0.35-2.36 | 0.84 | 0.71 | 0.32-1.56 | 0.39 |
| **DRR ≥first tertile vs. <third tertile** | 0.74 | 0.32-1.71 | 0.48 | 0.80 | 0.39-1.67 | 0.56 |
|  | **Multivariable PFS** | | | **Multivariable OS** | | |
|  | HR | 95%CI | P-value | HR | 95%CI | P-value |
| **DRR (continuous)** | 0.67 | 0.43-1.05 | 0.08 | / | / | / |
| Multivariable analysis adjusted for significant values in univariable analysis of the following: Eastern Cooperative Oncology Group performance status, histology subgroup, Memorial Sloan Kettering Cancer Center prognostic risk score, number of therapy lines, presence of sarcomatoid histology, T-stage, secondary malignancy, presence of liver metastasis and number of metastatic locations.  Adjustments in multivariable analyses for the intermediate group were performed for hepatic metastasis, histologic subgroups and sarcomatoid features, T-stage and lymph node metastasis in OS.  Adjustments in multivariable analyses for the poor group were performed for sarcomatoid features in PFS.  *Cut-off point for High and Low DeRitis ratio 1.58 | | | | | | |
