# Supplementary material for: Predictive value of De Ritis ratio in metastatic renal cell carcinoma treated with tyrosine-kinase inhibitors
Source: World J Urol. 2021 Mar 1;39(8):2977–85. doi: 10.1007/s00345-021-03628-2 (PMC8405478; doi:10.1007/s00345-021-03628-2)
Supplement: Supplementary file 2 — Supplementary file2 (DOCX 23 KB) [file 345_2021_3628_MOESM2_ESM.docx]

| Supplementary Table 2: Subgroup analysis according to clear-cell histology of different strata for De Ritis ratio (DRR) and its impact on progression-free (PFS) and overall survival (OS) | | | | | | |
| --- | --- | --- | --- | --- | --- | --- |
| **Clear-cell histology** | | | | | | |
|  | **Univariable PFS** | | | **Univariable OS** | | |
|  | HR | 95%CI | P-value | HR | 95%CI | P-value |
| **High DRR*** | 0.95 | 0.60-1.51 | 0.84 | 1.30 | 0.87-1.96 | 0.20 |
| **DRR (continuous)** | 1.09 | 0.83-1.43 | 0.55 | 1.28 | 0.99-1.66 | 0.06 |
| **DRR ≥1.0 vs. <1.0** | 1.20 | 0.82-1.76 | 0.35 | 1.40 | 0.98-2.00 | 0.06 |
| **DRR ≥1.1 vs. <1.1** | 1.02 | 0.70-1.48 | 0.93 | **1.56** | **1.10-2.21** | **0.013** |
| **DRR ≥1.2 vs. <1.2** | 0.93 | 0.63-1.36 | 0.70 | **1.44** | **1.01-2.04** | **0.042** |
| **DRR ≥1.3 vs. <1.3** | 1.00 | 0.68-1.47 | 1.00 | 1.38 | 0.97-1.96 | 0.08 |
| **DRR ≥1.4 vs. <1.4** | 0.97 | 0.65-0.44 | 0.87 | 1.22 | 0.84-1.76 | 0.29 |
| **DRR ≥1.5 vs. <1.5** | 0.92 | 0.60-1.42 | 0.71 | 1.25 | 0.85-1.84 | 0.26 |
| **DRR ≥1.6 vs. <1.6** | 0.95 | 0.60-1.51 | 0.84 | 1.30 | 0.87-1.96 | 0.20 |
| **DRR ≥1.7 vs. <1.7** | 0.91 | 0.55-1.51 | 0.72 | 1.19 | 0.77-1.84 | 0.44 |
| **DRR ≥1.8 vs. <1.8** | 1.24 | 0.74-1.08 | 0.43 | 1.52 | 0.97-2.40 | 0.07 |
| **DRR ≥1.9 vs. <1.9** | 1.24 | 0.69-2.21 | 0.48 | 1.55 | 0.94-2.56 | 0.08 |
| **DRR ≥2.0 vs. <2.0** | 1.32 | 0.66-2.62 | 0.43 | 1.62 | 0.91-2.88 | 0.10 |
| **DRR ≥median vs. <median** | 1.10 | 0.76-1.60 | 0.61 | **1.55** | **1.09-2.20** | **0.015** |
| **DRR ≥first tertile vs. <second tertile** | 1.42 | 0.89-2.26 | 0.14 | 1.36 | 0.87-2.10 | 0.173 |
| **DRR ≥first tertile vs. <third tertile** | 1.23 | 0.77-1.96 | 0.38 | 1.50 | 0.98-2.32 | 0.06 |
|  | **Multivariable PFS** | | | **Multivariable OS** | | |
| **DRR ≥1.1 vs. <1.1** | / | / | / | **1.53** | **1.04-2.24** | **0.029** |
| **DRR ≥1.2 vs. <1.2** | / | / | / | 1.31 | 0.89-1.91 | 0.17 |
| **DRR ≥median vs. <median** | / | / | / | **1.54** | **1.05-2.29** | **0.029** |
| **Non-clear-cell histology** | | | | | | |
|  | **Univariable PFS** | | | **Univariable OS** | | |
|  | HR | 95%CI | P-value | HR | 95%CI | P-value |
| **High DRR*** | 1.31 | 0.55-3.14 | 0.55 | 1.91 | 0.88-4.13 | 0.10 |
| **DRR (continuous)** | 0.84 | 0.52-1.34 | 0.46 | 0.94 | 0.59-1.51 | 0.81 |
| **DRR ≥1.0 vs. <1.0** | 0.71 | 0.32-1.61 | 0.42 | 0.70 | 0.35-1.40 | 0.31 |
| **DRR ≥1.1 vs. <1.1** | 1.00 | 0.47-2.14 | 1.00 | 0.91 | 0.46-1.78 | 0.78 |
| **DRR ≥1.2 vs. <1.2** | 0.87 | 0.40-1.86 | 0.71 | 0.88 | 0.45-1.73 | 0.71 |
| **DRR ≥1.3 vs. <1.3** | 0.68 | 0.30-1.50 | 0.34 | 0.70 | 0.35-1.43 | 0.33 |
| **DRR ≥1.4 vs. <1.4** | 1.05 | 0.48-2.32 | 0.90 | 0.82 | 0.40-1.69 | 0.60 |
| **DRR ≥1.5 vs. <1.5** | 1.24 | 0.55-2.80 | 0.60 | 1.25 | 0.62-2.52 | 0.53 |
| **DRR ≥1.6 vs. <1.6** | 1.31 | 0.55-3.14 | 0.55 | 1.91 | 0.88-4.13 | 0.10 |
| **DRR ≥1.7 vs. <1.7** | 1.07 | 0.43-2.68 | 0.89 | 1.76 | 0.80-3.87 | 0.16 |
| **DRR ≥1.8 vs. <1.8** | 0.90 | 0.34-2.41 | 0.84 | 1.69 | 0.75-3.84 | 0.21 |
| **DRR ≥1.9 vs. <1.9** | 1.10 | 0.41-2.93 | 0.85 | 1.58 | 0.67-3.71 | 0.30 |
| **DRR ≥2.0 vs. <2.0** | 0.91 | 0.31-2.67 | 0.87 | 1.36 | 0.55-3.35 | 0.50 |
| **DRR ≥median vs. <median** | 1.00 | 0.47-2.14 | 1.00 | 0.91 | 0.46-1.78 | 0.78 |
| **DRR ≥first tertile vs. <second tertile** | 0.85 | 0.31-2.32 | 0.75 | 0.68 | 0.28-1.68 | 0.41 |
| **DRR ≥first tertile vs. <third tertile** | 0.62 | 0.24-1.61 | 0.33 | 0.57 | 0.24-1.34 | 0.20 |
| Multivariable analysis adjusted for significant values in univariable analysis of the following: Eastern Cooperative Oncology Group performance status (ECOG), Memorial Sloan Kettering Cancer Center prognostic risk score (MSKCC), number of therapy lines, presence of sarcomatoid histology, T-stage, secondary malignancy, presence of liver metastasis and number of metastatic locations.  Adjustments in multivariable analyses for patients with clear-cell histology group were performed for ECOG, presence of sarcomatoid features, T-stage and lymph node metastasis as well as MSKCC in OS.  *Cut-off point for High and Low DeRitis ratio 1.58 | | | | | | |
