# Supplementary figures and images for: Predictive value of De Ritis ratio in metastatic renal cell carcinoma treated with tyrosine-kinase inhibitors
Source: World J Urol. 2021 Mar 1;39(8):2977–85. doi: 10.1007/s00345-021-03628-2 (PMC8405478; doi:10.1007/s00345-021-03628-2)

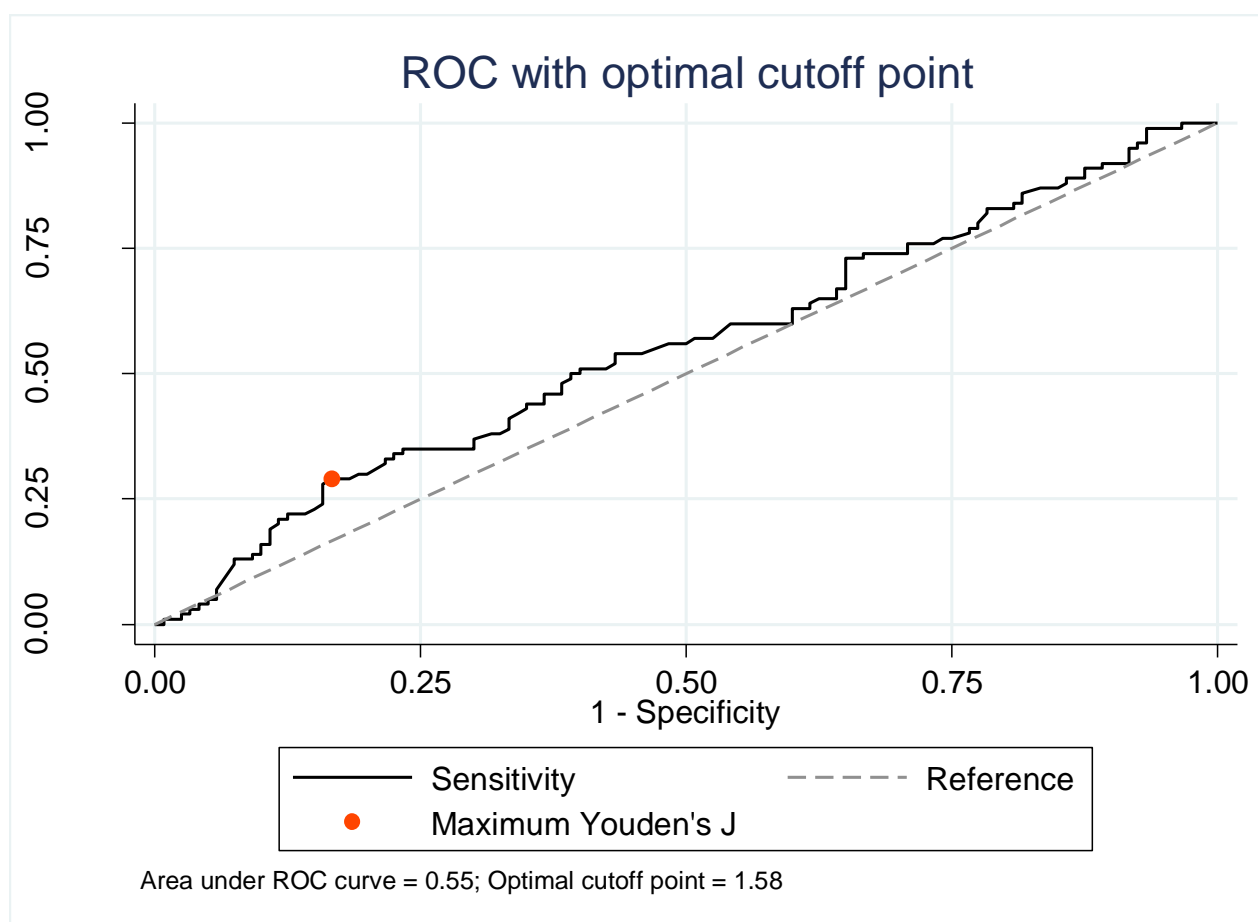

Supplementary Figure 1: Optimal Cutoff-point for De Ritis Ratio with Youden's J method

Supplement: Supplementary file 3 — Supplementary file3 (PDF 49 KB) [file 345_2021_3628_MOESM3_ESM.pdf]
