# Supplementary material for: Predictive value of De Ritis ratio in metastatic renal cell carcinoma treated with tyrosine-kinase inhibitors
Source: World J Urol. 2021 Mar 1;39(8):2977–85. doi: 10.1007/s00345-021-03628-2 (PMC8405478; doi:10.1007/s00345-021-03628-2)
Supplement: Supplementary file 4 — Supplementary file4 (PDF 56 KB) [file 345_2021_3628_MOESM4_ESM.pdf]

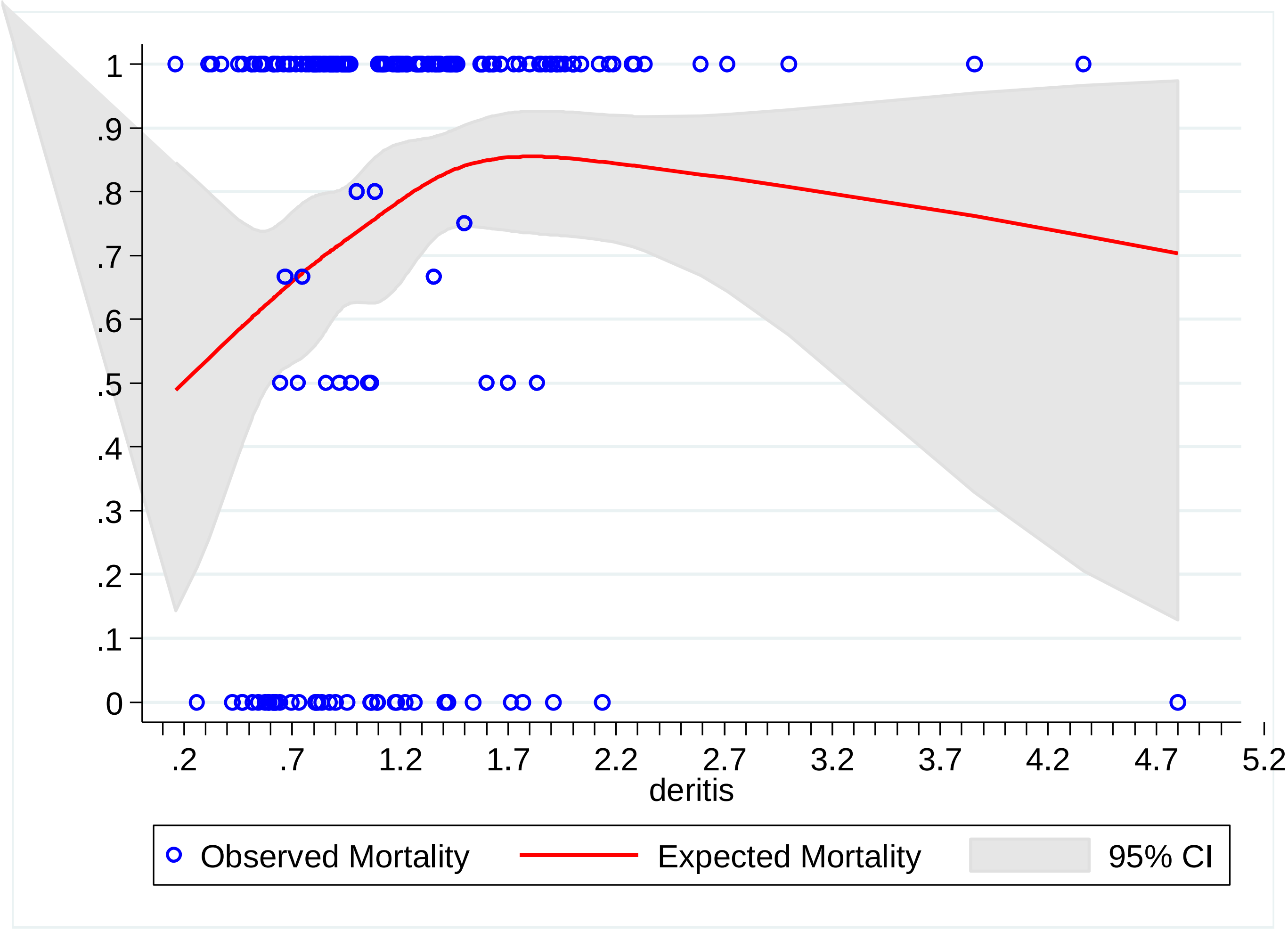


Supplementary Figure 2: Cubic spline regression of probability of Death in relation to the De Ritis ratio
