# Supplementary material for: Predictive value of De Ritis ratio in metastatic renal cell carcinoma treated with tyrosine-kinase inhibitors
Source: World J Urol. 2021 Mar 1;39(8):2977–85. doi: 10.1007/s00345-021-03628-2 (PMC8405478; doi:10.1007/s00345-021-03628-2)
Supplement: Supplementary file 5 — Supplementary file5 (PDF 73 KB) [file 345_2021_3628_MOESM5_ESM.pdf]

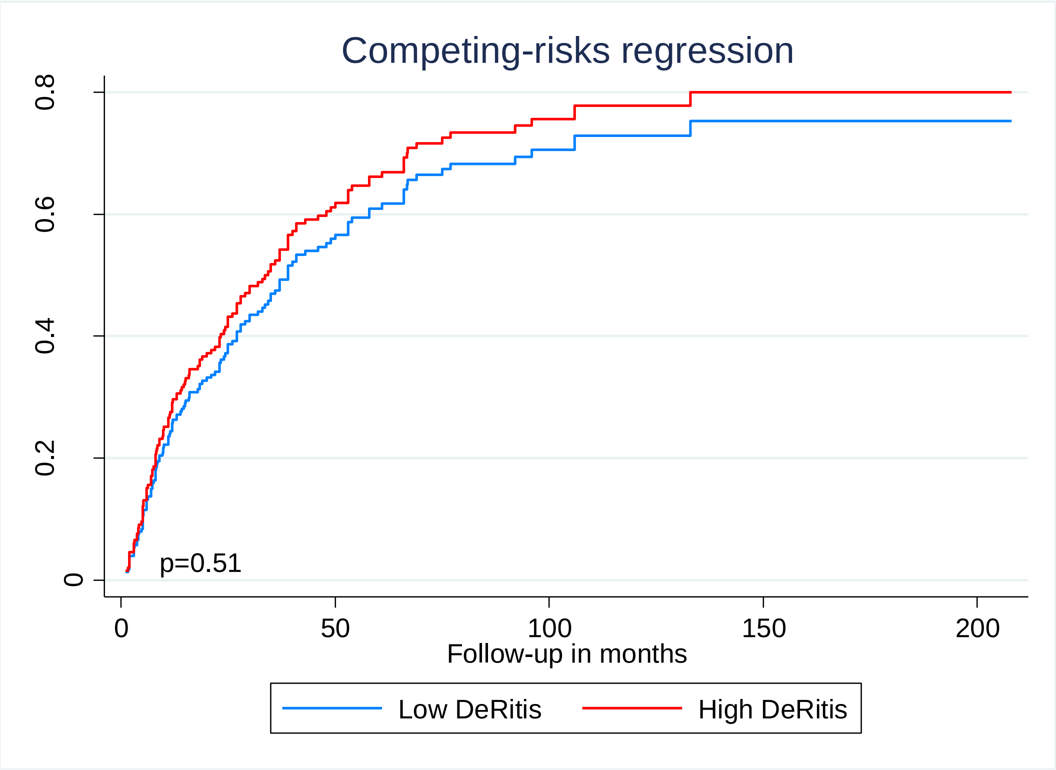

Supplementary Figure 3: Competing-risk regression for cancer-specific-/ and other-cause-mortality stratified by Deritis ratio ≥1.58 and <1.58.
